# Keep it cool: Unveiling the involvement of maize HEAT SHOCK FACTORS and CELLULOSE SYNTHASES in heat stress regulation

**DOI:** 10.1093/plcell/koae122

**Published:** 2024-04-17

**Authors:** Nitin Uttam Kamble

**Affiliations:** Assistant Features Editor, The Plant Cell, American Society of Plant Biologists; John Innes Centre, Norwich Research Park, Norwich, NR4 7UH, UK

With increasing threats of global warming, it is important to understand how heat is perceived and signalled inside the cells to activate mechanisms that protect plants from heat stress. It is known that a transcriptional cascade exists involving Heat Shock Factors (HSFs) to regulate the transcription of Heat Shock Protein (HSP) genes, which in turn regulate heat stress responses. In this issue, **Ze Li, Zerui Li, Yulong Ji, and colleagues (Li et al. 2024)** provide a thorough analysis of how HSFs regulate heat stress responses in maize.

The HSFs are categorized into 3 subfamilies, A, B, and C, based on their structures consisting of a deoxyribonucleic acid binding domain and an oligomerization domain (Scharf et al. 2012). Members of all these subfamilies have been linked to stress responses in a variety of plant species. To identify which HSFs are highly responsive to heat stress in maize, the authors performed deep transcriptome sequencing (RNA-seq) and weighted gene coexpression network analysis in maize seedlings. *ZmHSF20* of the HSFB subfamily was found to be one of the highly responsive genes to heat treatments. Functional characterization of nuclear-localized *ZmHSF20* via knockout or overexpression indicated that it is a negative regulator of heat stress tolerance. For instance, loss-of-function mutants were more resistant to heat because they accumulated less ROS with decreased ion leakage, whereas plants overexpressing *ZmHSF20* were more susceptible and showed increased ROS accumulation and ion leakage.

To identify target genes regulated by ZmHSF20, the authors performed DNA affinity purification sequencing (DAP-seq) of an adapter-ligated genomic DNA library and identified *ZmCesA* as a direct target of ZmHSF20. This was an interesting candidate for involvement in heat stress, as a previous study demonstrated that overexpression of *ZmCesA2* improved cold tolerance (Zeng et al. 2021). To investigate whether ZmHSF20 might regulate ZmHSFA family members induced by heat stress, the authors performed a yeast 1-hybrid assay and identified *ZmHSF4* as a direct target of ZmHSF20. Cleavage Under Targets and Tagmentation followed by qPCR and electrophoretic mobility shift assays confirmed that ZmHSF20 physically binds to *ZmCesA2* and *ZmHSF4* promoters. Using a firefly luciferase assay, they showed that ZmHSF20 binding directly represses the promoter activity of *ZmCesA2* and *ZmHSF4*.

Finally, overexpressing *ZmCesA2* and *ZmHSF4* led to an increased heat stress tolerance, mimicking the *Zmhsf20* loss-of-function mutant, and thus confirming that the negative effect of ZmHSF20 on heat shock resistance is due to a negative regulation of *ZmCesA2* and *ZmHSF4* expression. To further confirm this, the authors generated the *Zmhsf20-1 Zmhsf4-1* double mutant and demonstrated that *ZmHSF4* functions downstream of *ZmHSF20* in the heat stress response (see Figure). The authors further suggested ways in which more cellulose might improve heat tolerance by analyzing cellulose content and cell wall structure in lines having altered expression of *ZmHSF20*, *ZmCesA2*, and *ZmHSF4*.

**Figure.**
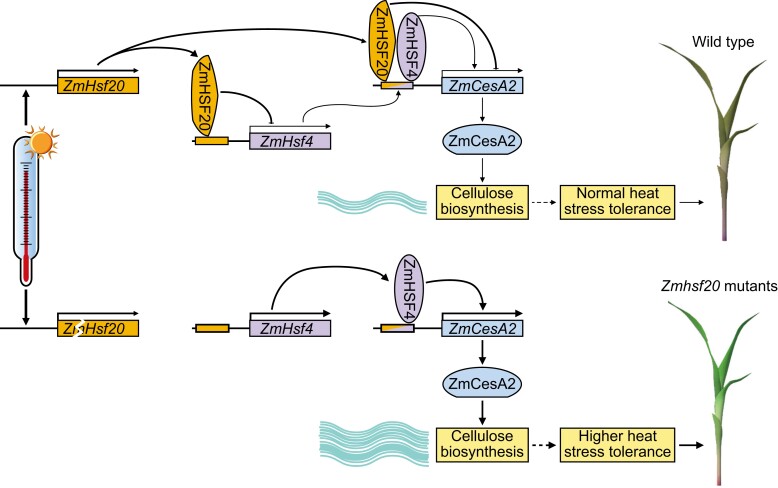
The heat stress response in wild-type maize (upper panel) involves the activation of *ZmHSF20*, which negatively regulates *ZmHSF4* and *ZmCesA2*, limiting cellulose biosynthesis. *Zmhsf20* mutant seedlings (lower panel) show enhanced tolerance to heat stress, as ZmHSF20-mediated repression of *ZmHSF4* and *ZmCesA2* is relieved. Image reprinted from Li et al. (2024), Figure 6.

In summary, this research sheds light on the mechanisms underpinning how HSFs, *ZmHSF20*, and *ZmHSF4* regulate heat stress responses in maize. In particular, ZmHSF20 was shown to negatively regulate the transcription of *ZmHSF4*, inhibiting its capacity to activate the transcription of downstream genes in heat responses. Further investigation will be needed to determine the exact connection between cell wall remodeling and the heat stress response. Understanding these mechanisms can have significant implications for improving plant growth under increasing threats of global warming.
